# Experimental Study Regarding Long Range LiDAR Capabilities in Sensing Safety Distance for Vehicle Application

**DOI:** 10.3390/s22155731

**Published:** 2022-07-31

**Authors:** Gabriel Popa, Marius-Alin Gheți, Emil Tudor, Ionuț Vasile, Ion-Cătălin Sburlan

**Affiliations:** 1 Rolling Stock Department from Faculty of Transport, University “Politehnica” of Bucharest, 060042 Bucharest, Romania; gabriel.popa@upb.ro (G.P.); marius.gheti@upb.ro (M.-A.G.); 2 Electrical Motors and Drives Laboratory, National Institute for Research and Development in Electrical Engineering ICPE-CA, 030138 Bucharest, Romania; ionut.vasile@icpe-ca.ro (I.V.); ion.sburlan@icpe-ca.ro (I.-C.S.)

**Keywords:** automated driving, autonomous vehicles, distance sensor, LiDAR sensor, long-distance sensor, safety distance, sensor sensitivity, tram safety

## Abstract

The safety of vehicles is one of the major goals of driving automation. The safety distance is longer for rail vehicles such as trams because of the adherence limitations of the wheel-to-rail system. The major issues of fixed frontal sensing are fake target detection, blind spots related to rail slopes, curves, and random changes in the target’s illumination or reflectivity. In this experimental study, distance measurements were performed using a scaled tram model equipped with a LiDAR sensor with a narrow field of view, under different conditions of illumination, size, and reflectivity of the target objects, and using different track configurations, to evaluate the effectiveness of such sensors in collision-avoidance systems for rail applications. The experimental findings are underlining the sensor’s sensitivity to fake targets, objects in the sensor’s blind spots, and special optical interferences, which are important for evaluating long-range LiDAR capabilities in sensing safety distance for vehicles. The conclusions can help developers to produce a dedicated colliding prevention system for trams and to identify the zones with high risk in the track where additional protection methods should be used. The LiDAR sensor must be used in conjunction with additional sensors to perform all the security tasks of an anti-colliding system for the tram.

## 1. Introduction

The automation of vehicle driving is a major goal of future transportation systems. One goal of driving automation is increasing traffic fluency while maintaining high safety. Regarding safety, most of the work is performed in developing autonomous vehicles based on several methods such as supervising the surrounding area for detection of imminent collision [1], precise location on map to define optimal route [2], automated interaction between cars for route optimization, and for automated braking system using time-to-contact detection [3].

Supervising the area is performed using optical means such as LiDAR and cameras. A LiDAR sensor has two main components: a light emitting circuit (infrared, LED or laser) and a receiver circuit [4].

Trams are special vehicles, and they can use some of the findings from autonomous drive and adopt some similar solutions for improved safety [5]. On-line GPS localization can impose some speed limitations based on the digital map information stored on board the vehicle [6,7]. Automated interaction between trams and signalization/traffic control signs is already implemented [8].

However, the sensing distance is below 30 m [9] as the braking system of the rubber wheels is more efficient than the wheel-rail systems of the tram.

The braking distance is longer for rail vehicles [10], and the maximum speed is limited to below 30 km/h in the mixed auto and rail traffic lines [11]. In the independent lines, the trams can be operated at speeds of 50 km/h or more, and the braking distance can be more than 90 m in several malfunction situations [12]. If the speed is 70 km/h, the safety distance is over 120 m [12]. In the special situation of the tram’s long braking distances, some precautions must be taken, such as the GPS map matching for active speed limitations [13] and implementation of long-range sensing for the automated braking system [14].

In railway applications, such as trams, onboard LiDAR sensors are used to determine the speed and the rail track quality, while safety measures are performed independently on ground equipment that advises the pilot about hindrances and prevents head-on, rear-end, and side-on collisions [15].

Similar work [16] was started by Siemens and presented an autonomous tram on the lines of Potsdam during Innotrans 2018, a prototype containing “multiple LiDAR, radar and camera sensors” [17], from which we can notice the wide-range LiDAR. Even if the tram is fully automated, a driver is still required to intervene when required. Two years later the system was commercially presented during a delivery announcement for Dusseldorf, in which they were presenting only cameras and radar [18]. Nowadays, they include in their communication an improved solution named “Collision Warning Assistant for Mainline” which can prevent accidents of the trams at speeds up to 45 km/h. Their presentation contains a LiDAR device able to perform detection of obstacles up to 600 m away. The research was performed on ICE trains and will be soon applied to trams, according to [19].

Alstom systems are described better in international patents and commercial presentations. A collision prevention system for trams is the hybrid solution presented in the patent [20], consisting of a fixed sensor system providing collision prediction to an onboard detection system that can control the automated braking system of the tram. This system was developed for the safety of the trams in the danger area of major crossroads where a full onboard system has bad visibility of the lateral roads. Patent [21] presents a long-range LiDAR sensor, without any description of the type, FoV, or range, focusing especially on the software of the automated signaling and braking of the tram. In the patent [22], there is a method for measuring the maximum range of the LiDAR sensors. Another patent [23] comprises four frontal sensors connected to a powerful computing unit that is performing three safety zones for the tram, using the actual speed of the tram. The sensors are not clearly defined, as they can be a selection of radar, camera, or LiDAR. Hence, the FoV of those sensors must be between 120° and 180°, specific for short-range sensors.

Alstom starts in 2017 with the autonomous stabling of a tram in the RATP depot, using LiDAR sensors, with excellent results. The project [24] is using also the wide FoV short-range LiDAR for low-speed automatic parking of the trams in the depot.

There are collision avoidance systems implemented in high-priced trams from Alstom and from Bombardier, such as ODAS [25] and COMPAS from AIT and MS, based on 3D cameras. The systems operate starting at 29.7 m from the obstacle.

In a commercial presentation of the Russian company Yandex [26], we can see that they are preparing a special LiDAR device able to adjust its scanning pattern to detect objects at 200 m distance; this device can be used for a prototype of a driverless tram in 2023 [27].

Another tram prototype was tested in Florence, implementing the safety system developed by Thales, and comprising multiple LiDAR, radar, cameras, and map localization techniques [28], with the goals of developing an integrated software architecture and testing the obstacle detection and avoidance functionality of ADAS (advanced driver assistance systems).

No academic references were found describing the application of long-range and narrow-FoV LiDAR for trams capable of detecting obstacles up to at least 90 m.

The Introduction section is presenting some of the most important papers regarding the LiDAR usage in the vehicular sensing, the Materials and Methods section is presenting the experimental model and the track and target configurations used for testing the issues. The Results section is presenting the measured data, which is analyzed in the Discussion section and leading to the findings presented in the Conclusion section.

## 2. Materials and Methods

The novelty of the research is to show how an onboard detection system that contains a single long-range LiDAR sensor behaves under certain track configurations. Our study was intended to show the limitations that this kind of long-range sensor has and evaluate if such a sensor can be used in an onboard tram safety system, in conjunction with several wide FoV sensors for low-speed operation.

The laboratory measurements we made were static, with the model standing still; however, the sensor and necessary electronics will be onboard a tram while it operates normally. We intend for the system to be used as a warning (stand-alone or redundant) solution for trams that operate on tracks without any kind of signaling system.

### 2.1. Description of Experimental Indoor Model

**Figure 1:**
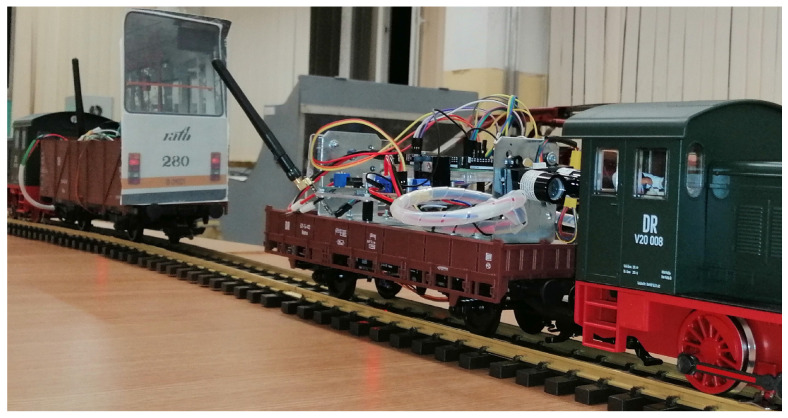
Scaled tram model for indoor testing of long-range LiDAR.

The experimental model was designed, executed, and tested by our team, with the goal of indoor-testing several types of long-range and narrow-field-of-view LiDAR sensors. Because we want to develop a special device for enhancing the safety of the trams, we started with a scaled train set class G (scale 1:22.5), as presented in Figure 1.

**Figure 2:**
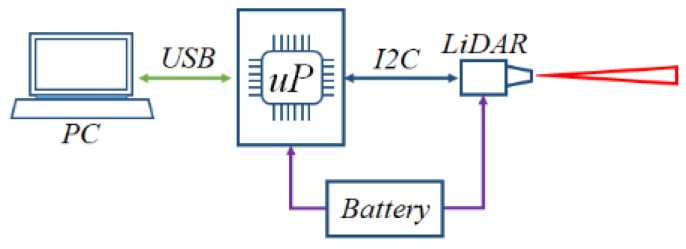
Experimental model basic diagram.

The basic diagram of the model is presented in Figure 2. A battery is used to power both a V3-Lite LiDAR sensor and an Atmega2560 microprocessor board that reads the information from the sensor via an I2C communication protocol.

The information from the microprocessor is then transmitted along a USB cable to a local PC for displaying and graphing the received data. Two values are read from the sensor, the measured distance *d* in centimeters and the signal strength *Ss*, which is a sensor internal value that has no units and is proportional to the amount of reflected light back to the sensor.

#### 2.1.1. Distance Detection Principle Using LiDAR

The measuring principle of the LiDAR is the ToF measuring because, knowing the ToF, one computes the distance between the sensor and the target using the equation:

*d* = *t_ToF_*·*c*/2 (1)

where *d* is the distance between the target and the LiDAR sensor, *t_ToF_* is the time of flight, and *c* is the speed of light.

#### 2.1.2. Long-Range and Narrow-Field-of-View LiDAR Sensor

For a LiDAR sensor to be able to detect objects placed at a 100 m distance, two improvements must be made: the powerful emitter diode must be equipped with a convergent optical lens with a high focal length and the sensitive receiver must have a divergent optical lens.

Furthermore, to improve the sensitivity, decrease the power consumption, and enhance the operational time of the sensor, some techniques of adjusting the diode’s emitted power with the returned signal’s strength are used. Therefore, for some producers, there is available the signal strength parameter for evaluation.

In Table 1 are some LiDAR sensors we have evaluated during this project and their main characteristics.

One can notice that the sensing area of those sensors is related to the distance between the sensor and the targets: the narrower the field of view the smaller the sensing area and the bigger the sensing distance. Most of the sensing areas are round, but specific sensors have an elliptical sensing area based on their special-shaped lens, making some of the sensors recommended for applications requiring more horizontal or vertical sensing area.

Another feature of these sensors is the return of an average value of the distance when the target object is not completely covering the sensing area, in which case the sensor will return a distance value that is an average of the distance to the target and the distance to the secondary object. This measurement can be noticed as an error; it must be detected by the software and it must be removed from the decision-making procedure. In Table 2 we explain the diameter of the FoV spot *d_FOV_* for different distances between the sensor and the target, as we will measure on the experimental model.

#### 2.1.3. Experimental Model Using LiDAR

Studying long-range LiDAR has a part that can be performed indoors, where several tests can be easily performed by using a scaled model. Models for a tramcar, a car, a pedestrian, and a pillar were prepared using a scale factor of 1:22.5, which is the scale of the model track that is being used. The sizes of the models, in terms of average height *h* and width *w* of real objects, are given by the following equations:

*h_tram* = 3200/22.5 = 142 mm (2)

*w_tram* = 1800/22.5 = 80 mm (3)

*h_car* = 1600/22.5 = 71 mm (4)

*w_car* = 1800/22.5 = 80 mm (5)

*h_person* = 1700/22.5 = 76 mm (6)

*w_person* = 400/22.5 = 18 mm (7)

*w_pillar* = 300/22.5 = 13.3 mm (8)

*w_parallel_line* = 3200/22.5 = 142 mm (9)

These dimensions do not consider things such as external mirrors, the pantograph, rooftop antennas, or similar irregularities in the shape of the objects.

### 2.2. Possible Measurement Errors Using Narrow-Field-of-View LiDAR

**Figure 3:**
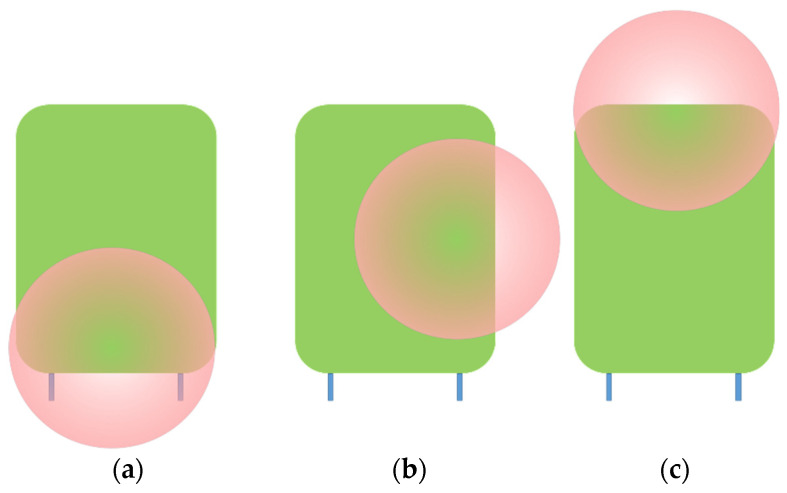
Possible misalignment of LiDAR *d_FOV_* with the target (**a**) uphill, (**b**) left curved, and (**c**) downhill.

Because of the narrow-field-of-view angle there is a possibility of a misalignment of the sensor with the target. In Figure 3 there are shown three possible mismatches between the sensor’s spot (see *d_FOV_* in Table 2) and the target.

To study these three cases, we prepared several experiments to test the limits of sensing with long-range LiDAR.

#### 2.2.1. Measurements in Curved Line

**Figure 4:**
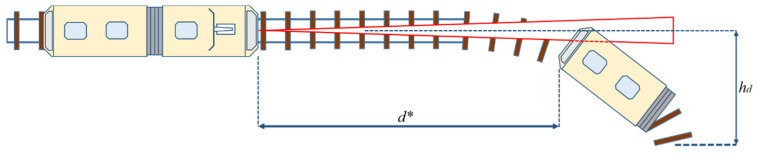
Horizontal misalignment LiDAR measurement.

For these experiments, we used the tramcar target. One end of the track was kept stationary while the other end of the track was displaced with the horizontal displacement *h_d_* measured from the center axis of the straight track, as shown in Figure 4.

The relation between the horizontal displacement *h_d_* and the radius of curvature of the track is given by Equation (10):

*R* = *d*^2^/*h_d_* (10)

#### 2.2.2. Slope Influence over Narrow-Field-of-View LiDAR

There are two possibilities: uphill slope and downhill slope. An important factor for these experiments is the height of the sensor above the track. Scaled height of the sensor axis is calculated in Equation (11).

*h_lidar_* = 1230/22.5 = 55 mm (11)

where 1230 mm is the minimum value of the height of the windshield of a tramcar.

**Figure 5:**
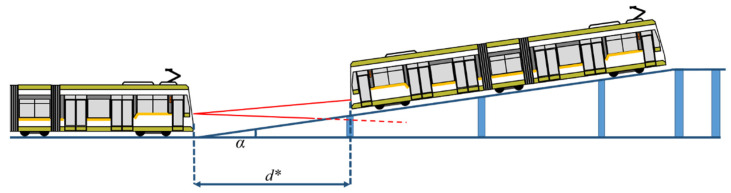
Uphill slope LiDAR measurement.

For the uphill slope experiment, the track was prepared using progressive height pillars to sustain the track at a certain slope (Figure 5) and we placed the tramcar target at increasing distances until the information from the LiDAR sensor did not match the reference distance.

**Figure 6:**
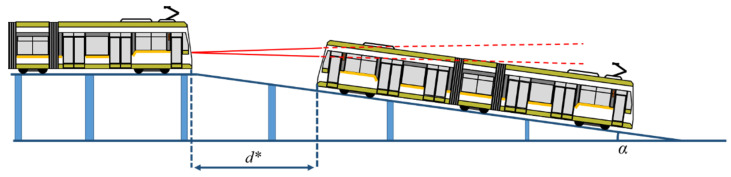
Downhill slope LiDAR measurement.

The downhill slope experiment was prepared in a similar way to the uphill experiment, using progressive pillars and placing the sensor at the highest point of the track, on a horizontal section of the track, as shown in Figure 6.

This experiment is important to determine the sensing capabilities of the narrow-field-of-view LiDAR when the tram approaches a tunnel under the level of the ground and has low visibility, the sensor being placed beneath the windshield of the tram.

#### 2.2.3. Fake Target Sensitivity

When using a LiDAR sensor, the provided distance information is related to the obstacle or to the targeted tram. This subsection aims to determine whether the pedestrian, the car, or the tram with the scaled dimensions as shown in Equations (2)–(8) are sensed as obstacles or fake targets to determine the limitations of measuring distance with a narrow-FoV LiDAR.

**Figure 7:**
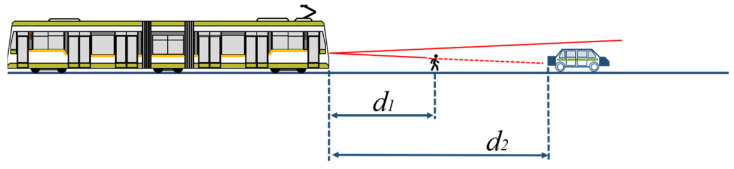
Pedestrian obstacle LiDAR measurement.

**Figure 8:**
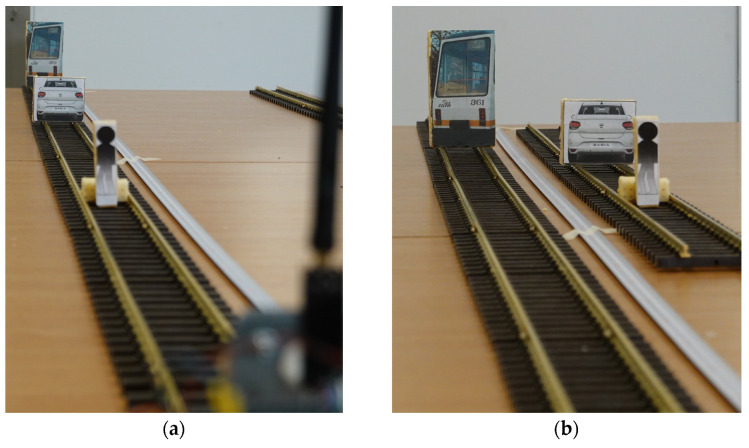
Fake targets used for indoor testing of long-range LiDAR.: (**a**) pedestrian model and/or car model placed between the tram equipped with sensor and the tram target; (**b**) pedestrian model and/or car model placed on the lateral line from the tram equipped with sensor and the tram target.

Measurements are to be made with smaller obstacles in front of the bigger obstacles, placed in front of the sensor at specific distances (*d*_1_ and *d*_2_ from Figure 7), on the same line (Figure 8a) or placed on a separated line (Figure 8b).

The distances for fake target measurements are resulting from the first tests performed, considering that the tram target should be placed at the distance where the FoV of the sensor is similar with the target dimensions. The fake targets must be placed at random distances between the sensor and the target.

#### 2.2.4. Target’s Behavior under Special Light Environment

**Figure 9:**
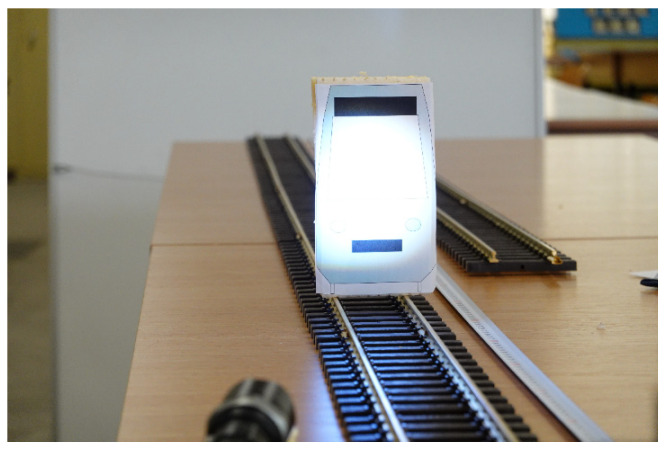
Powerful light on target object.

To test if the LiDAR measurement is influenced by certain light conditions a strobing LED light was shined on the target object (tram model) while the LiDAR was measuring the distance, as shown in Figure 9.

## 3. Results

### 3.1. Experimental Results for Horizontal Misalignment—Track Turning Left or Right

**Figure 10:**
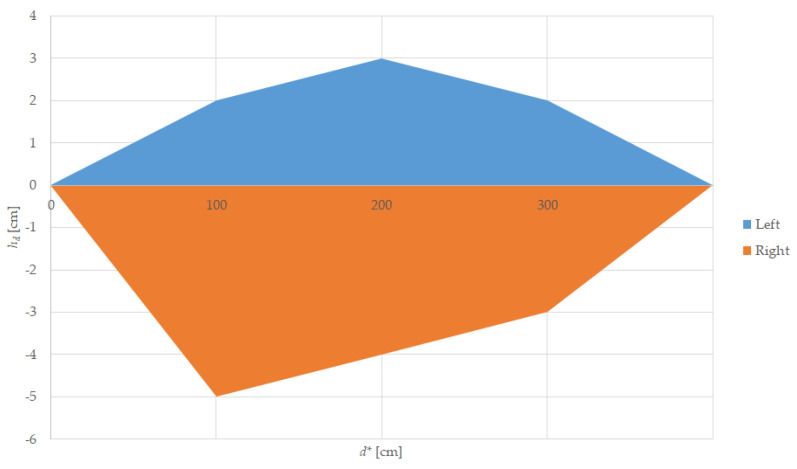
Horizontal displacement sensing area.

The results are presented in Table 3 for left displacement and Table 4 for right displacement, showing the reference distance in a straight line, d*, the displacement, *h_d_*, the LiDAR reported distance, *d*, and the signal strength *Ss*, as well as a graph for both left and right, as shown in Figure 10.

### 3.2. Experimental Results for Vertical Misalignment—Track Going Uphill and Downhill

#### 3.2.1. Uphill Slope

**Figure 11:**
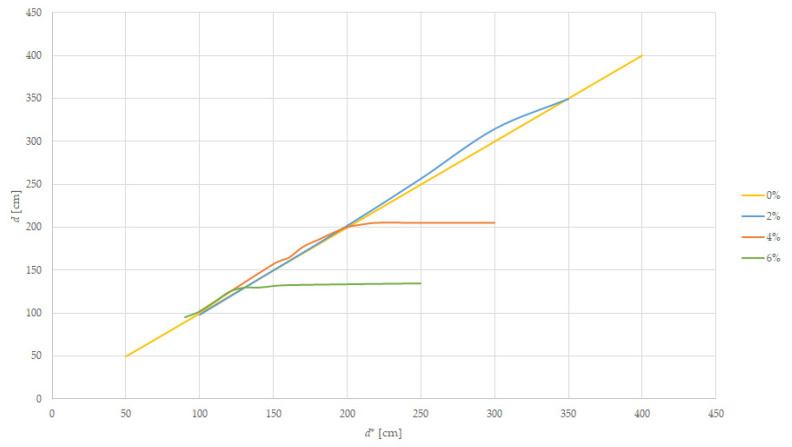
Distance measurement on uphill slope.

For this experiment, we prepared the track using pillars and we placed the tramcar target, as computed in Equations (2) and (3), at increasing distances, d*, with the result shown in Table 5 for a tramcar target and Table 6 for a car target. Figure 11 is presenting the measured value versus the target’s frontal displacement when the target is moved uphill. Those measurements are influenced by the height between the LiDAR sensor and the track, as computed in Equation (8).

The measurements are relevant while the LiDAR measured distance is equal to the distance between the sensor and the target. For better results we performed multiple tests near the value where the returned distance remains unchanged, meaning that the target is not in the FoV of the sensor, but the existing track is returning the light of the LiDAR. Those values are 200 cm on a 4% slope and 130 cm on a 6% slope. In Table 6, we repeated the measurements on the uphill modeled track using the car model as a target.

The results from Table 5 and Table 6 are the same because the FoV will aim at the base of the vehicle, which is moving far and up from the fixed FoV of the LiDAR, and the height difference between tram and car becomes irrelevant.

#### 3.2.2. Downhill Slope

**Figure 12:**
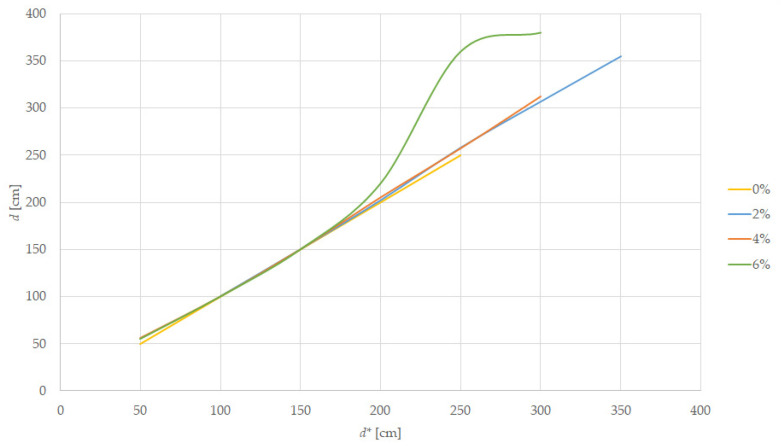
Distance measurement on downhill slope.

A scaled downhill track was prepared using pillars and we placed the tramcar target at increasing distances, *d*, until the information from the LiDAR sensor did not match the reference distance d*, with the result shown in Table 7 and summarized in Figure 12.

The errors appear at 200 cm for the 2% slope, at 300 cm for the 4% slope, and 150 cm for the 6% slope; the sensed distance being the one to the wall, the target is no longer in the FoV of the sensor. The sensibility of the sensor is decreased because the sensor is placed at 55 mm above the track. The car target was not sensed by the LiDAR at any distance in the downhill experiment.

### 3.3. Experiments Using Fake Targets

This experiment presented in Figure 8a involves placing two different targets on the track at different distances, *d*_1_, and *d*_2_ (as in Figure 7), from the sensor and observing the measurement, *d*, reported by the sensor to see how the targets influence the reading. Results are shown in Table 8.

These experiments are supporting the affirmation that the major obstacles, as the car and the pedestrian are, will not interfere with the latter target of the tram, and the returned values of distance will be, most of the time, the distance to the closest obstacle from the sensor.

The experiment with fake targets on a lateral line was using distance computed from Equation (9), placing a tram, a car, and a pedestrian on a parallel line placed at 142 mm distance between the axis, and a pillar of 13.3 mm placed half-way. None of these objects were interfering with the LiDAR distance reported value.

### 3.4. Experimental Results in Different Lighting Conditions

For this experiment we set up the tramcar target at varying distances and use an LED strobing light to see if the LiDAR measurement is influenced by this change in lighting conditions. The results are shown in Table 9.

The LiDAR reported value *d* and the signal strength *Ss* are not influenced by the external light.

### 3.5. Experimental Results with Different Target Color

For this experiment, we used two different tramcar targets that are colored differently, with light and dark colors. The targets were placed at different distances, *d_light_* and *d_dark_*, and a comparison between them is shown in Table 10.

Different colors of the target have minor influence over the measured values.

## 4. Discussion

The model used for this work is in accordance with the parameters of the full-scale tram, including the FoV of the LiDAR sensor, which is identical to the one used in this paper, the sensor height position on the tram (*h_lidar_*), the dimensions of the target tram, and the sensing distance will be scaled respecting the selected G scale with a ratio of 1:22.5. The distances presented in the discussion will be normal distances at 1:1 scale.

A dedicated software was developed, which reports in a simple way the distance to the target and the strength of the signal. Unfortunately for the analyzed sensor the signal strength is an internal value that cannot be used as additional information to differentiate two objects at the same distance. When the measurements are returning correct values, the signal strength has 126–140 values, while errors are accompanied by weaker signal values of 98–112.

The tram operates with maximum speed mostly on separated and straight lines, where the LiDAR sensor proves to be very efficient at distances up to 90 m. On a major curved line where the target tram will be misaligned horizontally, the sensor’s small FoV will not cover the vehicles placed outside this detection area and additional measures must be taken into account, such as a 30 km/h speed limiter in track curves with a radius less than 100 m. This is a must, especially for the tram itself, to prevent derailment caused by centrifugal force.

For horizontal displacement we can note two observations. The first one is that the left and right displacements from which the distance measurement is returning incorrect values are not the same, especially at 22.5 m distance, because the sensor itself has a horizontal displacement between the emitter and the receiver lens.

The second note is that, at important distances, errors appear at smaller lateral displacements. One explanation is that the LiDAR spot is larger and most of the target is not covered by the FoV, as presented in Table 2. For example, at 67.5 m distance the horizontal displacement from which we have errors is between 0.45 m and 0.675 m, a value close to the FoV diameter from Table 2 (2.65 cm representing 0.6 m).

If the tram is running on a track going upward (an ascending slope) the sensing distance will be diminished because the sensor will measure the distance to the mid-range targets or the distance to the track. From the measurements on the 4% slope the sensing distance falls below 45 m. In this case, because the braking distance will be smaller when braking uphill, there are no measures to be taken.

In the downhill running case, the sensor on the tram will see only close-range targets, missing some small-size obstacles such as the cars and the pedestrian. In this case, the best solution is to reduce speed to below 30 km/h because smaller obstacles are not detectable before the slopes of more than 4%. Additional care must be considered when going downhill, especially because the braking distance will be bigger.

The sensor with a small FoV will be insensitive to fake targets on parallel lines, including cars, pedestrians, and aerial line poles.

In addition, the sensor information can be perturbed when a small-size obstacle is placed on the same line with the target, especially because the sensor will report an average value of the distance. The solution to avoid fake alarms from the sensor is that the signal must be validated for a specified time (in our case 0.5 s) prior to setting the alarm on.

The mounting position of the LiDAR sensor on the tram is very important to the sensing capabilities because, if we are placing the sensor to low, it will not cover a long distance, especially with a slow ascending slope, as proved in Section 3.2.1. A sensor placed higher on the tram will not be sensitive enough for medium size obstacles, such as pedestrians or cars, as demonstrated when measuring the downhill distance to cars, which were not sensed starting with an equivalent of 25 m real distance.

When changing the illumination of the target or the illumination of the environment there were no important changes in the measurements of distance and only small influences can be noticed on the signal strength, thus being a normal operation of such a sensor.

Future work is focused on tests with the already developed full-scale TRL4 experimental model of a long-range narrow-FoV distance detector, which will be tested outdoors on a separated track. That model can be tested afterward in real traffic conditions.

For the automated braking system for the tram, a three-level braking system must be developed, based on the LiDAR information. The three levels are, according to the ADAS standards from the auto industry and braking standards for rail vehicles, first a warning signal of 1.5 s maximum rolling time, followed by an automated braking with deceleration of 1 m/s^2^ covering 1.5 s, and a complete emergency brake with more than 2.8 m/s^2^ until complete stop. The driver can interfere with this system by moving the master controller handle on the “Brake” section, a feature needed to avoid excessive use of the brake because of false alarms.

## 5. Conclusions

A long-range LiDAR sensor with a narrow FoV is recommended to be used by the automated braking systems of trams, especially trams that must be operated with high speed on long and separated lines. This type of LiDAR sensor is not influenced by the lighting conditions, by the color of the target, or by the objects that are outside the detection area.

The sensor position on the tram influences the distance measurement: the higher position of the sensor on the tram, the longer the sensing distance, but the lower the performance when the tram is going downhill.

The field of view and the sensing distance of the sensor are the most important parameters taken into consideration when selecting a long-range LiDAR sensor for trams. Based on the measurements performed and presented in this paper, we recommend a sensor with an FoV of 0.5° and 90 m minimum detection range for use in an anti-colliding system for trams. We hope that the LiDAR sensor producers will focus on such sensors that can improve the long-range sensitivity of the ADAS systems for trams and for general-use vehicles, as well.

Due to the limitations of the long-range LiDAR, a complete prevention system must contain additional sensors like wide-FoV LiDAR or radar sensors for collision prevention, backed up with cameras for obstacle and traffic sign recognition.

If possible, a secondary short-range wide-FoV LiDAR can be placed in front of the tram, closer to the ground, to check whether there are some close-range obstacles in front of the tram like small pedestrians or animals, fallen objects, or small objects.

A frontal camera can be used to improve the functionality of the automated brake system, especially when used to protect against frontal colliding at high speed. A complementary safety solution is using the speed limiter of the tram, implemented based on GPS localization of portions of the rail that represent blind spots for the LiDAR sensor.

## Figures and Tables

**Table 1:** Main characteristics of several narrow-field-of-view LiDAR.

Characteristic	TF03	Leddar-Vu8	V4-Lite	V3-Lite	TFmini-S
Range [m]	180	185	10	40	12
Resolution [cm]	1	1	1	1	1
FoV [°]	0.5	0.3	4.77	0.5	1

**Table 2:** Field of view for the TF03 LiDAR for different distances to target.

**Distance [cm]**	100	200	300	400
**FoV [°]**	0.5	0.5	0.5	0.5
***d_FOV_*** **[cm]**	0.87	1.75	2.62	3.5

**Table 3:** Left horizontal displacement experimental results with tramcar target.

*d*_1_*	*h_d_*_1_	*d*_1_	*Ss*_1_	*d*_2_*	*h_d_*_2_	*d*_2_	*Ss*_2_	*d*_3_*	*h_d_*_3_	*d*_3_	*Ss*_3_
[cm]	[cm]	[cm]		[cm]	[cm]	[cm]		[cm]	[cm]	[cm]	
100	0	97	126	200	0	202	126	300	0	307	140
	1	98	126		1	202	126		1	308	140
	2	97	126		2	202	140		2	311	140
	3	103	140		3	203	140		3	329	140
	4	410	98		4	228	112		4	395	112
	5	410	98		5	415	126		5	415	140

**Table 4:** Right horizontal displacement experimental results with tramcar target.

*d*_1_*	*h_d_*_1_	*d*_1_	*Ss*_1_	*d*_2_*	*h_d_*_2_	*d*_2_	*Ss*_2_	*d*_3_*	*h_d_*_3_	*d*_3_	*Ss*_3_
[cm]	[cm]	[cm]		[cm]	[cm]	[cm]		[cm]	[cm]	[cm]	
100	0	97	126	200	0	202	126	300	0	307	140
	1	97	126		1	202	126		1	308	140
	2	98	126		2	202	126		2	308	140
	3	97	126		3	202	126		3	311	140
	4	96	126		4	208	126		4	331	126
	5	97	126		5	348	70		5	380	112
	6	415	112		6	415	140		6	406	140

**Table 5:** Uphill vertical track experimental results with tramcar target on track.

Uphill Slope	d*	*d*	*Ss*
	[cm]	[cm]	
2%	100	98	126
	150	150	126
	200	202	140
	250	257	140
	300	315	140
	350	350	126
4%	100	102	126
	150	157	140
	160	164	140
	170	177	140
	180	185	140
	190	193	140
	200	200	140
	210	203	140
	220	205	126
	250	205	126
	300	205	126
6%	90	95	140
	100	102	140
	110	113	140
	120	125	140
	130	130	140
	140	130	154
	150	132	154
	160	133	145
	200	134	145
	250	135	145

**Table 6:** Uphill vertical track experimental results with car target on track.

Uphill Slope	d*	*d*	*Ss*
	[cm]	[cm]	
2%	100	98	126
	150	147	140
	200	203	140
	250	258	140
	300	312	140
	350	401	140
4%	100	97	126
	140	140	140
	150	151	140
	160	167	140
	170	175	140
	180	186	140
	190	194	140
	200	200	140
	210	203	140
	220	207	126
6%	90	91	126
	100	103	126
	110	114	140
	120	125	140
	130	131	140
	140	133	140
	150	135	140
	160	134	140

**Table 7:** Downhill vertical track experimental results with tramcar target on track.

Downhill Slope	d*	*d*	*Ss*
	[cm]	[cm]	
2%	100	100	126
	150	150	140
	200	202	140
	250	258	140
	300	307	140
	350	355	140
4%	50	56	126
	100	100	112
	150	150	126
	200	205	126
	250	257	126
	300	312	140
6%	50	55	126
	100	100	112
	150	150	126
	200	220	126
	250	360	98
	300	370	98

**Table 8:** Different objects in the sensor field of view experimental results.

Targets	*d*_1_	*d*_2_	*d*	*Ss*
	[cm]	[cm]	[cm]	
Tram and car	300	200	212	140
Tram and car	300	100	100	126
Tram and pedestrian	300	200	210	140
Tram and pedestrian	300	100	99	126
Car and pedestrian	300	200	210	112
Car and pedestrian	300	100	99	126
Car and pedestrian	200	100	101	126

**Table 9:** Different lighting conditions experimental results.

d*	*d*	*Ss*
[cm]	[cm]	
100	101	126
200	202	140
300	300	140
400	402	126

**Table 10:** Different color target experimental results.

d*	*d_light_*	*d_dark_*	*Ss*
[cm]	[cm]	[cm]	
100	97	102	126
150	148	152	126
200	200	207	126
250	255	257	140
300	307	309	140

## Data Availability

Not applicable.
